# Flow Cytometry To Assess Cerebrospinal Fluid Fungal Burden in Cryptococcal Meningitis

**DOI:** 10.1128/JCM.03002-15

**Published:** 2016-02-25

**Authors:** James E. Scriven, Lisa M. Graham, Charlotte Schutz, Thomas J. Scriba, Robert J. Wilkinson, David R. Boulware, Graeme Meintjes, David G. Lalloo, Britta C. Urban

**Affiliations:** a Liverpool School of Tropical Medicine, Liverpool, Merseyside, United Kingdom; b Wellcome Trust Liverpool Glasgow Centre for Global Health Research, Liverpool, United Kingdom; c Clinical Infectious Diseases Research Initiative, Institute of Infectious Disease and Molecular Medicine, University of Cape Town, Cape Town, South Africa; d Department of Medicine, University of Cape Town and Groote Schuur Hospital, Cape Town, South Africa; e South African TB Vaccine Initiative, Institute of Infectious Disease and Molecular Medicine and Department of Paediatrics and Child Health, University of Cape Town, Cape Town, South Africa; f Department of Medicine, Imperial College, London, United Kingdom; g Francis Crick Institute, Mill Hill Laboratory, London, United Kingdom; h Department of Medicine, University of Minnesota, Minnesota, USA

## Abstract

Fungal burden in the cerebrospinal fluid is an important determinant of mortality in cryptococcal meningitis, but its use in aiding clinical decision making is hampered by the time involved to perform quantitative cultures. Here, we demonstrate the potential of flow cytometry as a novel and rapid technique to address this issue.

## TEXT

Cryptococcal meningitis (CM) remains one of the commonest causes of meningitis in sub-Saharan Africa and a significant cause of death among persons with HIV-1 infection (1, 2). Cerebrospinal fluid (CSF) fungal burden is an important determinant of mortality but requires quantitative culture, a time-consuming process taking several days, limiting its usefulness as a clinical aide in decision making (3). Recent *in vitro* work using broth dilutions of Cryptococcus neoformans demonstrated a very close association between the number of cryptococci counted using a flow cytometer and quantitative culture (4). This raises the possibility that flow cytometry is a useful technique to rapidly assess fungal burden in patients with cryptococcal meningitis. However, no studies have examined this technique on *ex vivo* samples. We addressed this by performing flow cytometry counting of cryptococci in CSF samples from patients with HIV-1-associated CM and compared these counts with measurement of fungal burden using quantitative CSF culture. This study formed part of a larger body of work aimed primarily at examining the CSF immune response in CM.

**FIG 1:**
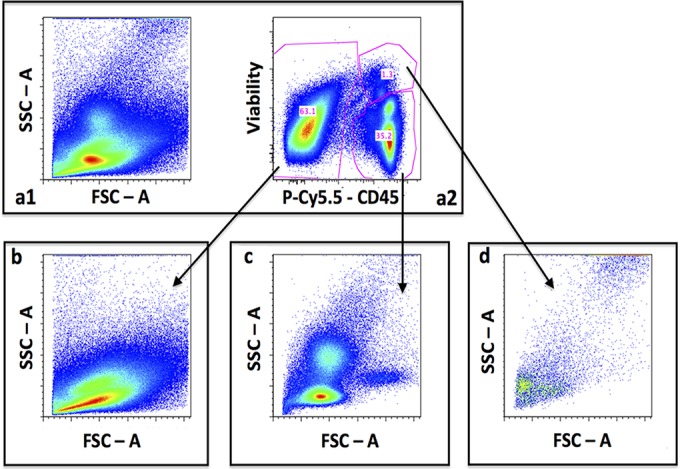
CSF flow cytometry gating CSF cells (forward scatter [FSC] and side scatter [SCC]), showing a poor definition of cell subsets due to cryptococci (a1), CD45 and LIVE/DEAD stain are used to separate cells (a2), FSC-SSC plot of cryptococci (b), FSC-SSC plot of CSF leukocytes (c), and FSC-SSC plot of dead CD45^+^ cells.

A prospective cohort study was conducted in Cape Town, South Africa. All participants provided written informed consent; surrogate consent was obtained from the next of kin for patients with impaired consciousness. Ethical approval was obtained from the University of Cape Town (Cape Town, South Africa) and Liverpool School of Tropical Medicine (Liverpool, United Kingdom) research ethics committees. HIV-infected persons age ≥18 years with a first episode of cryptococcal meningitis (diagnosed by antigen test or culture) were enrolled within 48 h of diagnosis, and lumbar puncture performed to measure the cerebrospinal fluid (CSF) opening pressure. CSF fungal burden was assessed with quantitative culture (5) and cryptococcal antigen (CrAg) titer (CrAg lateral flow assay [LFA]; Immy, USA), as previously described (6). The volume of remaining CSF was measured and the cells pelleted using centrifugation; this was incubated on ice with an amine viability dye (Aqua; Invitrogen) and anti-CD45-PECy5.5 (BioLegend) and then at room temperature with flow-activated cell sorting (FACS) lysing solution (BD Biosciences), protected from light at all times. Cells were fixed using 2% paraformaldehyde and analyzed within 24 h on a BD LSRFortessa flow cytometer using the FACSDiva software (BD Biosciences). A forward scatter threshold of 5,000 was used to avoid including any debris in counting; the sample was acquired in its entirety to allow a calculation of cell counts. Compensation was performed using species-appropriate compensation beads (BD Biosciences; Invitrogen). Data were analyzed using Flow Jo version 9.5.3 (Tree Star software) (Fig. 1). Cryptococci were defined as CSF cells negative for the panleukocyte marker CD45. The counts for the whole sample were divided by the CSF volume to obtain Cryptococcus counts per milliliter of CSF. Statistical analyses were performed using Stata version 12 (StataCorp).

Sixty HIV-infected patients with cryptococcal meningitis were enrolled, with a median CD4 count of 34 cells/μl of CSF. CSF samples were available for 58 participants, 36 of whom had not received any amphotericin B prior to enrollment. The median CSF volume collected for flow cytometry was 7 ml (interquartile range [IQR], 4.5 to 8 ml). The median fungal burden was 4.74 log_10_ CFU/ml of CSF (IQR, 3.5 to 5.75 log_10_ CFU/ml), as measured by quantitative culture, and 4.53 log_10_ Cryptococcus yeasts per ml of CSF (IQR, 3.33 to 5.21 log_10_ CFU/ml of CSF), as measured by flow cytometry. The median CrAg LFA titer was 1:8,000 (IQR, 2,000 to 16,000).

**FIG 2:**
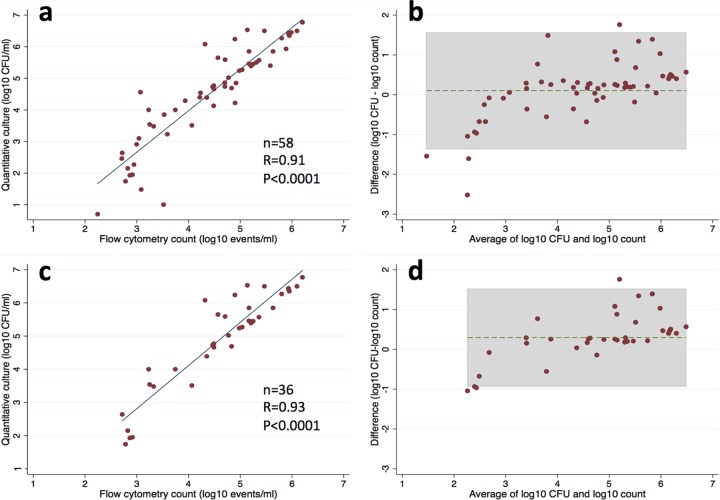
(a) Scatterplot demonstrating association between flow cytometry counting, and quantitative culture. (b) Bland-Altman plot showing good agreement between flow counting and quantitative culture (gray area indicates the 95% limits of agreement). (c) Association between flow counting and quantitative culture among participants who had not received antifungal therapy before CSF sampling. (d) Bland-Altman plot showing agreement between the two techniques, limited to participants who had not received antifungal therapy before CSF sampling (gray area indicates the 95% limits of agreement).

The cryptococcal counts measured by flow cytometry were strongly correlated with both quantitative culture (Pearson's *r* = 0.91; *P* < 0.0001) (Fig. 2a) and CrAg titer (Spearman rho = 0.75; *P* < 0.0001). Linear regression showed that the quantitative culture result could accurately be predicted from flow cytometry counting (log_10_ CFU/ml of CSF = 1.31 × log_10_ flow count − 1.28; *R*^2^ = 0.82; *P* < 0.0001). The agreement between flow cytometry counting and quantitative culture was also assessed using a Bland-Altman plot (Fig. 2b). This showed good agreement between these two measurements, with a mean difference of −0.1 log_10_ CFU/ml of CSF and only 6.9% (4/58) of values outside the 95% limits of agreement. These outlying values were found mainly among participants with low fungal burdens (<500 CFU/ml of CSF), whereas flow cytometry counting produced values that were approximately 1- to 2-log_10_ CFU/ml of CSF higher. Similarly strong correlation and agreement between the two measurement techniques were also noted when analysis was restricted to the 36 participants who had not received any antifungal therapy prior to CSF sampling (Pearson's *r* = 0.93; *P* < 0.0001 (Fig. 2c); the mean difference was −0.30 log_10_ CFU/ml of CSF, with 11.1% (4/36) of the values outside the limits of agreement (Fig. 2d).

These findings suggest that flow cytometry has the ability to provide a rapid and accurate measurement of fungal burden in persons with HIV-associated cryptococcal meningitis. If combined with a cryptococcal viability stain (as previously demonstrated *in vitro* [4]), flow cytometry could also be used to assess the response to treatment.

Due to the well-recognized toxicity of amphotericin B (7), there is considerable interest in short-course regimens, particularly in those patients with low fungal burden (8). The results from quantitative culture are not available in a timely enough manner to inform clinical decision making, but the rapidity of the result obtained from flow cytometric cryptococcal counting potentially overcomes this problem. This might allow for a reduction in drug toxicity, cost, and duration of hospitalization. Although this technique does require access to a flow cytometer, suitable machines are available in many laboratories in sub-Saharan Africa, where they are used to measure CD4 counts. In areas where they are not available, a rapid assessment of fungal burden might be possible using quantitative microscopy (4).

There were a number of limitations to this study. No cryptococcus-specific stain was used to identify cryptococci; instead, they were assumed to be any CD45^−^ cell found in the CSF. Given that all participants had laboratory-confirmed cryptococcal meningitis, a lysis buffer was used to ensure that no erythrocytes remained in the CSF, and host leukocytes were excluded using the panleukocyte marker CD45; we feel this is a reasonable assumption and that our results are valid. However, to be a fully robust clinical assay, an anticryptococcal stain would ideally be incorporated into the panel and the assay validated on CSF samples from patients who do not have CM. This would have the additional benefit of improving the accuracy of flow cytometric counting at low fungal burdens, where debris or miscellaneous cells may have interfered with the counting process. In addition, we examined only the use of flow cytometric counting to assess fungal burden at baseline and did not assess changes on antifungal therapy. Future work should aim to incorporate a cryptococcal viability marker to address this issue, as previously assessed *in vitro* (4).
